# miR-29 regulates Tet1 expression and contributes to early differentiation of mouse ESCs

**DOI:** 10.18632/oncotarget.10751

**Published:** 2016-07-21

**Authors:** Yanhua Cui, Ting Li, Dehua Yang, Song Li, Weidong Le

**Affiliations:** ^1^ Center for Translational Research on Neurological Diseases, the First Affiliated Hospital, Dalian Medical University, Dalian, China; ^2^ Key Laboratory of Stem Cell Biology, Institute of Health Sciences, Shanghai Institutes for Biological Sciences, Chinese Academy of Sciences, Shanghai, China; ^3^ Collaborative Innovation Center for Brain Science, the First Affiliated Hospital, Dalian Medical University, Dalian, China

**Keywords:** Tet1, 5hmC, embryonic stem cells, miR-29 family

## Abstract

The ten-eleven translocation-1 (Tet1), which converts 5-methylcytosine (5mC) to 5-hydroxymethycytosine (5hmC), plays important roles in many important biological processes, such as mouse embryonic stem cells (ESCs) maintenance. However, the mechanisms for Tet-1 regulation remain largely unknown. Here we showed that miR-29 family (miR-29a, miR-29b and miR-29c) can directly repress Tet1 expression. We found that Tet1 was highly expressed and 5hmC was presented at relatively high levels in mouse ESCs, but the levels of both Tet1 and 5hmC were reduced during the early differentiation of ESCs. On the contrary, miR-29 level was increased in this process. ESCs stably transfecting with miR-29 precursors showed lower levels of Tet1 protein and 5hmC. Furthermore, we demonstrated that miR-29 overexpression selectively affected cell lineage markers and skewed ESC differentiation, which was similar in Tet1 knockdown ESCs. Our results indicate that miR-29 is a direct regulator of Tet1 in mouse ESCs.

## INTRODUCTION

DNA methylation at the 5-position of cytosine (5-methylcytosine, 5mC) is essential for numerous biological processes, including gene regulation, genomic imprinting, X chromosome inactivation and mammalian development [1–4]. DNA methylation is relatively stable and the mechanisms of DNA demethylation in mammals have remained elusive until recently. The ten-eleven translocation (TET) family of proteins (Tet1, Tet2 and Tet3), which oxidize 5mC to 5-hydroxymethylcytosine (5hmC) and further to form 5-formylcytosine and 5-carboxylcytosine [5–8], are thought to be involved in active and/or passive DNA demethylation [9]. Moreover, previous studies have demonstrated that this TET-catalyzed 5hmC regulates gene expression in differentiating colonocytes and colon cancers [10], and the levels of TET and 5hmC are dramatically reduced in human breast, liver, lung, pancreatic and prostate cancers [11].

Interestingly, while the reaction intermediate 5hmC is strongly depleted in human cancers, 5hmC has been shown to be abundant in embryonic stem cells (ESCs) and non-cancerous tissues. Tet1 protein and 5hmC are present in high levels in mouse ESCs and adult brain, suggesting a role in epigenetic control of these cells and tissues [12–15]. Indeed, Tet1 has an important role in mouse ESCs maintenance and functions to regulate the lineage differentiation potential of ESCs [16, 17]. Acute depletion of Tet1 impairs LIF/Stat3 signaling and results in loss of ESC identity [18]. Genome-wide analyses of Tet1 and 5hmC distribution by high-throughput sequencing (ChIP-seq) found that Tet1 has dual functions in transcriptional regulation in mouse ESCs, it can bind to both actively transcribed H3K4me3-only genes and PRC-repressed CpG-rich genes, thus can associate with either activated or repressed transcriptional states [19–21]. In adult brain, Tet1 promotes 5mC hydroxylation, activates DNA demethylation, and is critical for neuronal activity-regulated gene expression and memory formation [22–24]. Tet1 deficient mice exhibit impaired hippocampal neurogenesis accompanied by poor spatial learning and memory [25]. Consistently, 5hmC exists at high levels in mouse ESCs, however, its level significantly decreases after mouse ESC differentiation [26, 27]. Moreover, during development or differentiation from ESCs to terminally differentiated neurons, 5hmC levels are dynamically changed at specific gene bodies and/or promoters [28]. Considering the important roles of Tet1/5hmC in regulation of many genes, it will be of a great need to elucidate how this catalytic process is controlled.

MicroRNAs (miRNAs) are a class of small, noncoding RNAs of ∼22-nucleotides that regulate gene expression at the post-transcriptional level [29]. These molecules mostly destabilize target mRNAs or suppress translation by binding to complementary sequences in the 3′ untranslated region (3′UTR) [30]. miRNAs play roles in diverse processes including cell proliferation, cell differentiation, apoptosis and development [31–35]. Recent studies have suggested that miR-29 family could target at Tet1 and is involved in the pathogenesis of human malignancies [36–38]. However, whether miR-29 is involved in regulation of Tet1 in ESCs remains as a subject of further investigation.

In this study, we screened a panel of miRNAs which were predicted to target Tet1 and found that miR-29 family members (including miR-29a, miR-29b and miR-29c) can target Tet1 at 3′UTR and repress its expression directly. We found that Tet1 was highly expressed in mouse ESCs and decreases during the early differentiation, and was partially regulated by miR-29. We further documented that miR-29 overexpression in ESCs caused a similar phenotype as Tet1 knockdown. These data suggest miR-29 is a direct regulator of Tet1 and may provide potential strategies for cancer diagnosis and therapy.

## RESULTS

### miR-29a/b/c target Tet1 via direct binding to 3′UTR *in vitro*

**Figure 1:**
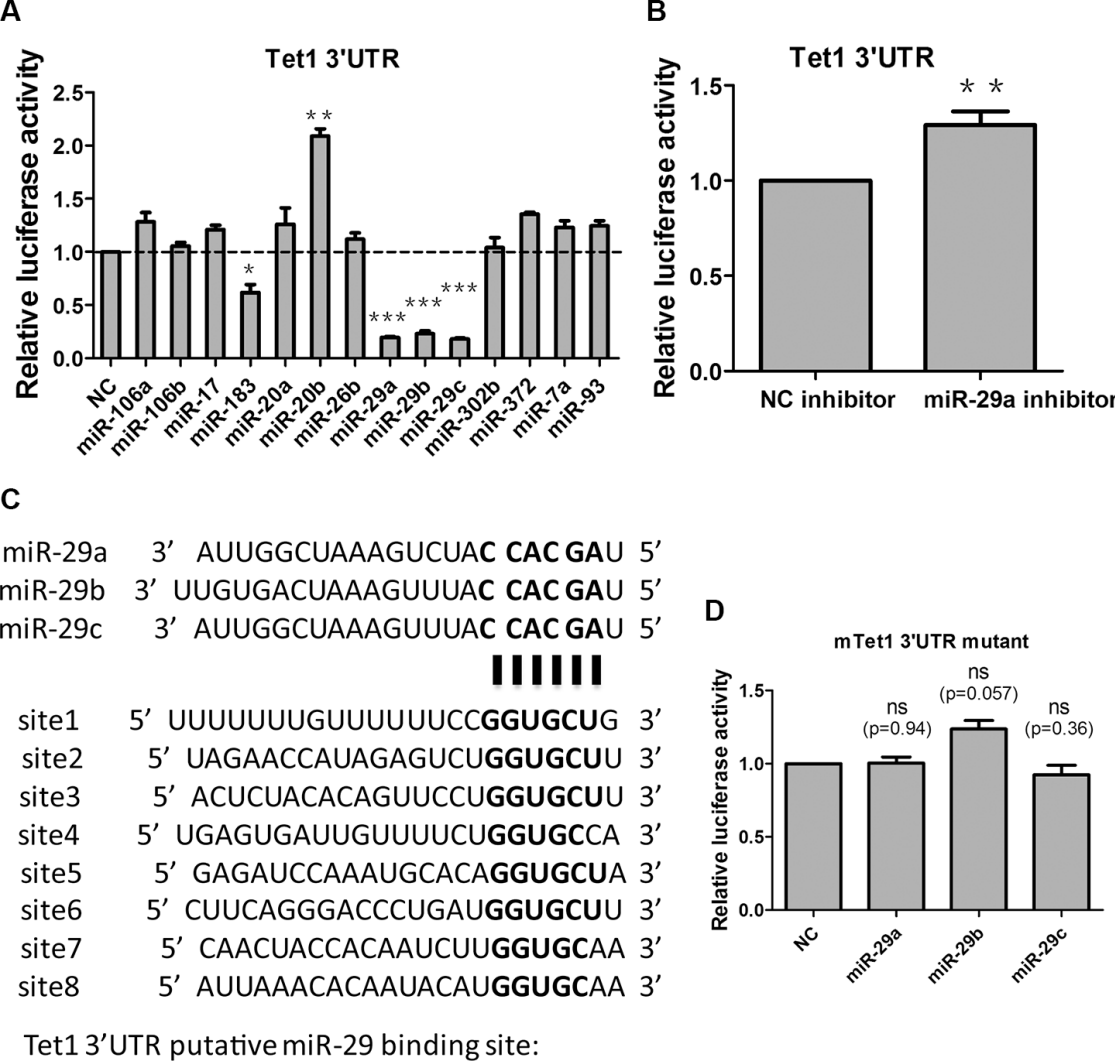
miR-29a/b/c target Tet1 via direct binding to 3′UTR *in vitro* (**A**) Screen the miRNAs which are predicted to target Tet1 with PicTar software via dual luciferase reporter assay. A 1698bp mouse Tet1 3′UTR was cloned into a luciferase reporter vector, and cotransfected with different miRNA mimics to 293T cells. Luciferase activity was measured 48 h after transfection. Data are shown as mean ± SEM (*n* = 3). (**B**) miR-29a inhibitor increased the relative luciferase activity of vectors containing Tet1 3′UTR. Data are shown as mean ± SEM (*n* = 6). (**C**) 8 putative miR-29a/b/c target sites are present in Tet1 3′UTR. (**D**) miR-29a/b/c cannot inhibit the relative luciferase activity of vectors containing Tet1 3′UTR with all 8 putative binding sites mutated. Data are shown as mean ± SEM (*n* = 3). ns means no significance, *p < 0.05, **p < 0.01, ***p < 0.001.

To investigate whether miRNAs are involved in Tet1 regulation, we analyzed Tet1 3′UTR using the target prediction software PicTar [39–41]. We found that there were a few miRNAs predicted to target Tet1: miR-106a, miR-106b, miR-17, miR-183, miR-20a, miR-20b, miR-26b, miR-29a, miR-29b, miR-29c, miR-302b, miR-372, miR-7a and miR-93. Then we performed dual luciferase reporter assay to identify which miRNAs are true regulator of Tet1 *in vitro*. miRNAs mimics or negative control (NC) mimics and a psiCheck2 luciferase reporter plasmid containing the whole length of Tet1 3′UTR were co-transfected into HEK-293T cells, and the luciferase activity were measured. We found that miR-29 family (miR-29a, miR-29b and miR-29c) and miR-183 significantly inhibited the relative luciferase activity while miR-20b increased the relative luciferase activity (Figure 1A). As miR-29a/b/c exhibit the highest inhibition effect among screened miRNAs, we focused on this miRNA family for further study. At the same time, miR-29a inhibitor increased the relative luciferase activity slightly but significantly compared to NC inhibitor (Figure 1B). Tet1 3′UTR contains 8 putative binding sites to miR-29a/b/c (Figure 1C), we mutated “seed region” in these sites and found that miR-29 mimics were unable to reduce the relative luciferase activity (Figure 1D). Taken together, all these *in vitro* data showed that miR-29 family can target Tet1 via direct binding to its 3′UTR.

### miR-29a/b/c levels increase while Tet1 reduces during the early differentiation of mouse ESCs

**Figure 2:**
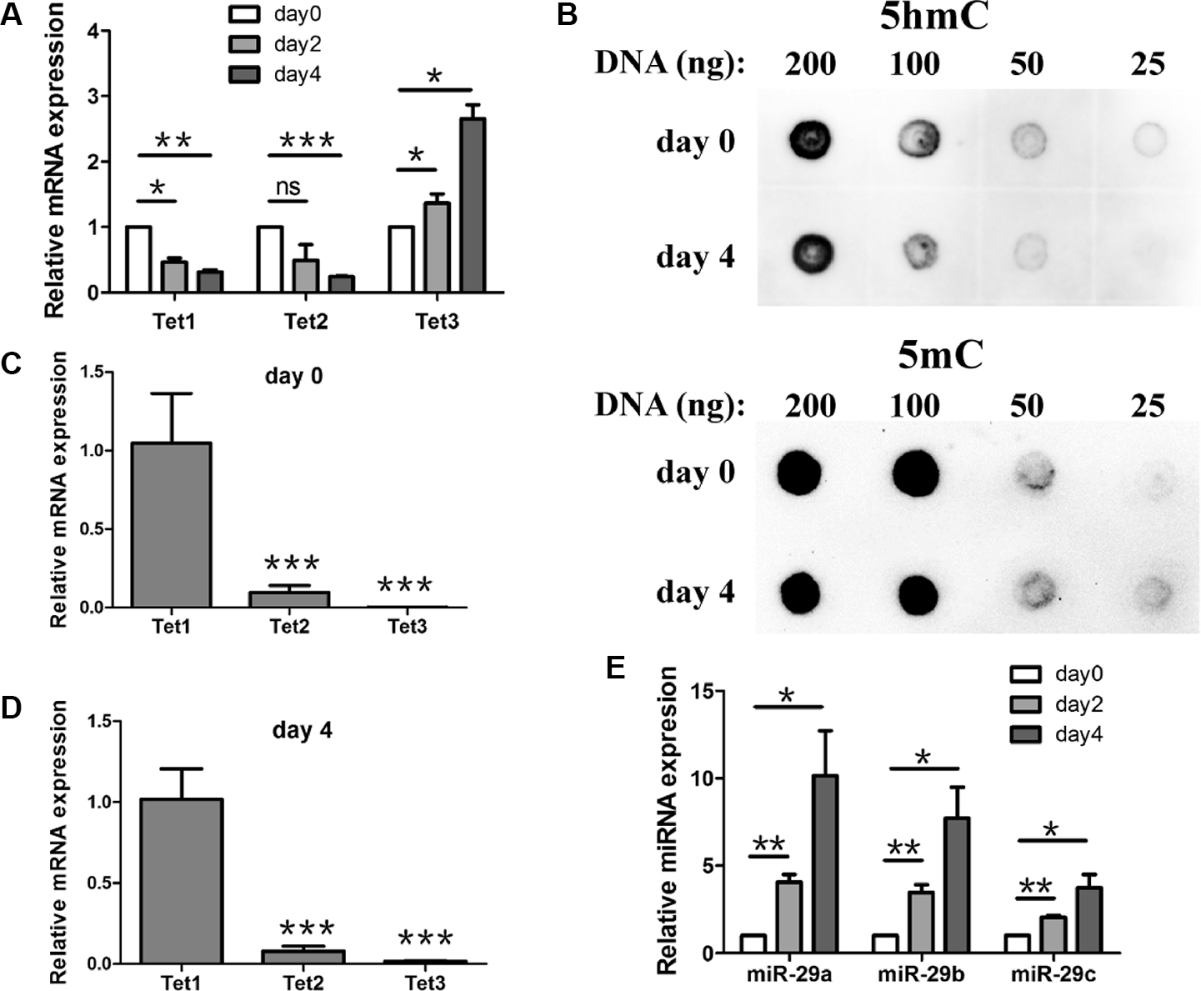
Expression profile of Tet1/2/3, miR-29a/b/c and the change of 5hmC during mouse ESCs early differentiation (**A**) RT-qPCR analysis of Tet1/2/3 expression during ESC early differentiation. ESCs were differentiated spontaneously as embryoid bodies (EBs) for different days and cells were collected for gene expression analysis. Data are shown as mean ± SEM (*n* = 3). (**B**) DNA dot blot assay showed that 5hmC level (up panel) was reduced after 4 days differentiation compare with undifferentiated ESCs. 5mC levels (down panel) were used as an internal control. (**C** and **D**) Expression levels of Tet2 and Tet3 in EBs differentiated for 0 day (C) and 4 days (D) were relative to Tet1 (set to 1) respectively. (**E**) RT-qPCR analysis of miR-29a/b/c expression during ESC early differentiation. ESCs were differentiated spontaneously as embryoid bodies (EBs) for different days and cells were collected for gene expression analysis. Data are shown as mean ± SEM (*n* = 3). ns means no significance, *p < 0.05, **p < 0.01, ***p < 0.001.

To elucidate the significance of miR-29 targeting Tet1, we examined Tet1 mRNA and miR-29a/b/c levels during the early differentiation of mouse ESCs. Tet1 mRNA and 5hmC were reported to present high levels in mouse ESCs [17]. When ESCs were differentiated spontaneously as embryoid bodies (EBs) for different days, Tet1 and Tet2 mRNA levels declined rapidly, while Tet3 mRNA levels increased slowly (Figure 2A). Meanwhile, 5hmC level reduced moderately (Figure 2B, up panel), and 5mC level was used as a loading control (Figure 2B, down panel). For all of the three Tet proteins that could generate 5hmC, we compared the transcript levels of the genes to see which protein contributes to the change of 5hmC level mainly. We found that Tet1 transcripts were present at highest level in both undifferentiated ESCs and EBs differentiated for 4 days (Figure 2C and 2D). Tet2 transcripts in undifferentiated ESCs were about 10-fold less abundant than Tet1, while Tet3 transcript levels were the lowest, about 1000-fold less abundant than Tet1 (Figure 2C). When ESCs were differentiated as EBs for 4 days, Tet2 transcripts were still about 10-fold less abundant than Tet1, while Tet3 transcript levels were about 100-fold less abundant than Tet1 (Figure 2D). So Tet3 upregulation at day 4 could not compensate for the reduced Tet1 and Tet 2. The Tet1 transcripts were presented at highest level at both, day 0 and day 4, we attributed the decrease of 5hmC level mainly to Tet1 downregulation. In the contrary, miR-29a/b/c levels increased during this process (Figure 2E). Because miR-29a/b/c bound and repressed Tet1 directly *in vitro*, we suggested that Tet1 was negatively regulated by miR-29a/b/c during ESCs early differentiation.

### miR-29 negatively regulates Tet1 expression in mouse ESCs and promotes the upregulation of trophoblast lineages markers

**Figure 3:**
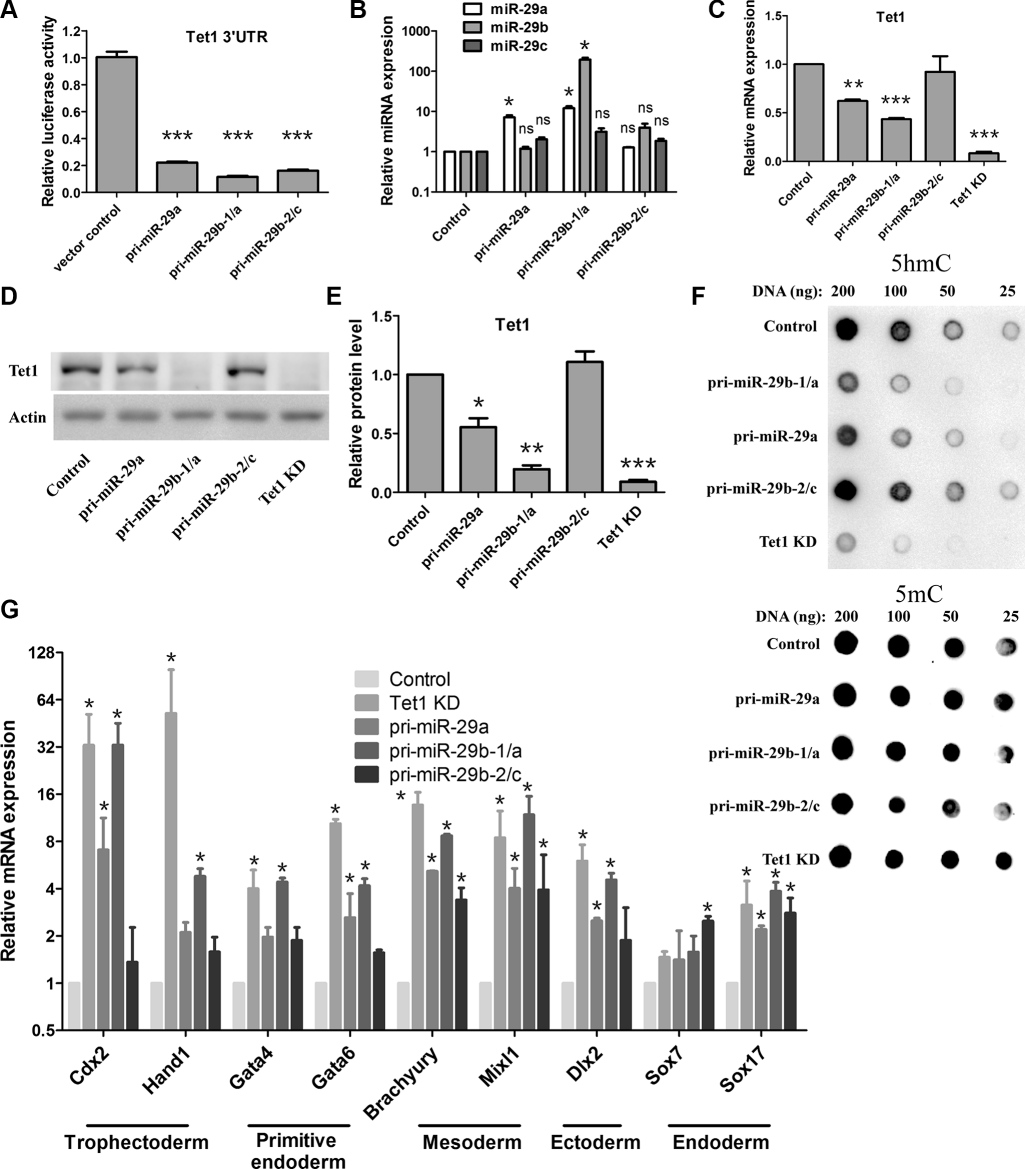
miR-29 negatively regulates Tet1 expression in mouse ESCs and promotes upregulation of trophoblast lineages markers (**A**) miR-29 precursors' activities were confirmed by dual luciferase reporter assay. Data are shown as mean ± SEM (*n* = 3). (**B**) RT-qPCR analysis of miR-29a/b/c in ESC lines stably transfected with different miR-29 precursors. The expression levels of each microRNA in vector control cells are set as 1. Data are shown as mean ± SEM (*n* = 3). (**C**) RT-qPCR analysis of Tet1 mRNA in ESC lines stably transfected with different miR-29 precursors. Tet1 knockdown (Tet1 KD) cells were used as positive control. The expression levels in vector control cells are set as 1. Data are shown as mean ± SEM (*n* = 3). (**D** and **E**) Western blot analysis of Tet1 proteins in different ESCs (C). Actin served as a loading control. The density of the bands was analyzed by Image J software and expressed as fold of control (E). (**F**) DNA dot blot analysis of 5hmC levels (up panel) in different ESCs. 5mC levels (down panel) were used as an internal control (**G**) RT-qPCR analysis of the expression of various cell lineage markers in different ESCs. The expression level in vector control cells is set as 1. Data are shown as mean ± SEM (*n* = 3). ns means no significance, *p < 0.05, **p < 0.01,***p < 0.001.

To further verify the repressive impacts of miR-29 on Tet1 expression in mouse ESCs, we established cell lines stably transfecting with miR-29 precursors. miR-29 family members are encoded by two gene clusters in the genome: miR-29a/b-1 and miR-29b-2/c [42, 43]. We constructed three miR-29 overexpressed vectors, pri-miR-29a, pri-miR-29a/b-1 and pri-miR-29b-2/c, and confirmed their activities by dual luciferase reporter assay (Figure 3A). ESCs overexpressing these miRNA precursors were generated respectively, and Tet1 knockdown cells expressing a lentiviral knockdown short hairpin RNAs (shRNAs) for Tet1 was established as a positive control cell line.

We firstly determined the miR-29 family member expression in these cell lines. We found that the cell line overexpressing pri-miR-29a/b-1 showed the highest level of miR-29 family members (Figure 3B), while the cell line overexpressing pri-miR-29b-2/c showed a moderately high level of miR-29 family members compared to the control cell line, but there was no statistical difference between the two cell lines (Figure 3B).

Tet1 mRNA and protein levels in these cell lines were determined by real-time quantitative PCR (RT-qPCR) and western blot assay. As expected, the Tet1 mRNA levels were reduced in three miR-29 overexpressing ESCs (Figure 3C), with pri-miR-29a/b-1 showed the best effect on Tet1 repression. Similar to the Tet1 knockdown ESCs, Tet1 protein level in pri-miR29a/b-1 expressing ESCs was almost undetectable. In contrast, ESCs expressing pri-miR-29b-2/c showed a nearly normal Tet1 protein level as in vector control ESCs (Figure 3D and 3E), which were consistent with the relative transcriptional activity of these constructs in ESCs. As 5hmC could be produced by Tet1, we examined 5hmC levels in these cell lines as well. Consistent with the reduced Tet1 protein levels, ESCs expressing pri-miR-29a and pri-miR-29a/b-1 also showed significant reduction of 5hmC levels, while pri-miR-29b-2/c expressed cells kept a normal 5hmC levels as the control cells (Figure 3F, up panel), 5mC levels were used as an internal control (Figure 3F, down panel).

Previous studies report that Tet1 functions to regulate the lineage differentiation potential of ESCs, Tet1 loss-of-function in ESCs results in developmental skewing towards endoderm/mesoderm and trophoblast lineages [16, 17]. To verify whether miR-29 overexpression could cause a similar effect on ESCs, we analyzed several markers of early differentiation via RT-qPCR. Consistent with previous studies, knockdown of Tet1 in ESCs resulted in a selective upregulation of trophoblast lineage markers, including Cdx2, Hand1, Gata4, Gata6 (Figure 3G). Consistent with Tet1 knockdown, ESCs expressing pri-miR-29a/b-1 showed a similar expression pattern of these early differentiation markers (Figure 3G), which further confirmed that miR-29 could negatively regulate Tet1 expression in ESCs.

## DISCUSSION

Here we show that miR-29 family members can negatively regulate Tet1 expression via direct binding to its 3′UTR, which was also reported in two papers recently [36, 44]. Additionally, our present study further elucidated the biological significances of this relationship between miR-29 and Tet1. We firstly demonstrated the direct regulation of Tet1 by miR-29 *in vitro*, and then we investigated the role of miR-29 in mouse ESCs and confirmed that Tet1 was repressed by miR-29 during the early differentiation of mouse ESCs. miR-29 overexpressed ESCs showed a reduced level of 5hmC and upregualted levels of several early differentiation markers, which was consistent with Tet1 loss-of-function ESCs. However, whether miR-29 mediated Tet1 suppression is required for ESC differentiation is not elucidated here. To answer this question, miR-29 knockout ESC should be established. Unfortunately, since there are 3 members in miR-29 family, it will be difficult and laborious to knockout all these miRNAs using traditional gene targeting strategy, such as homology recombination mediated gene targeting. To our surprise, a new technology using the CRISPR/Cas system which allows the one-step generation of cell lines on animals carrying mutations in multiple genes was developed recently [45], which may prove as a promising tool for miR-29a/b/c knockout and help answering the above mentioned question.

Previous research has reported that miR-29 could directly target both DNA methyltransferases 3A and 3B and is involved in DNA methylation [46]. In this study, we found that miR-29 negatively regulated Tet1 and promoted the generation of 5hmC, which is considered to be an intermediate product in the process of DNA demethylation. These results are not conflicted, as miRNAs may regulate hundreds of target mRNAs and often fine-tunes the expression of target genes. Whether miR-29 promotes DNA methylation or demethylation may be determined by amount of miR-29 in a cell or interaction with other regulators.

Recent studies have found that Oct 4 could be replaced by Tet1 during induced pluripotent stem cell (iPSC) induction [47], we compared the expression of miR-29 between fibroblast and ESCs and found that miR-29 was expressed highly in fibroblasts but decreased heavily in ESCs (data not shown), which was contrary to the change of Tet1. As some certain miRNAs have been proved to promote reprogramming of somatic cells to pluripotency more efficiently [48], whether controlling the expression of Tet1 via miR-29 may help to iPSC induction requires further investigation.

In addition, miR-29a and miR-29b were also reported to function as tumor suppressors in leukogenesis [49–51]. Meanwhile, Tet1 has been identified as a fusion partner of the MLL gene in acute myeloid leukemia [52] and also involves in some kinds of leukemia [53, 54]. Moreover, recent research findings have suggested that miR-29 may directly regulate Tet protein and is involved in cancer progression. Takayama K et al. have revealed a novel divergent function of miR-29 as a crucial epigenetic regulator that represses TET2 in cancer progression [55]. Consistently, Lin et al. have also elucidated the roles of feedback of miR-29-Tet1 downregulation in hepatocellular carcinoma development, Thus, miR-29-Tet signaling may serve as potential target for the prognosis of cancers developing. In addition, the novel epigenetic approaches for inhibiting miR-29 or modifying TET-mediated signaling pathways may have important implications for cancer therapy.

In summary, our study proves miR-29 as a direct regulator of Tet1 and provides possible mechanisms on how miR-29 and Tet1 interact and play bio-functions. Our data also highlight miR-29 as a potential therapeutic target in treating Tet1-related human diseases.

## MATERIALS AND METHODS

### Cell culture

The mouse E14Tg2A ESCs were maintained on 0.1% gelatin-coated dishes in DMEM containing 15% FBS, LIF (1,000 U/ml), GlutaMAX, nonessential amino acids, penicillin/streptomycin and β-mercaptoethanol under feeder-free conditions. HEK-293T cells (obtained from the Cell Bank at the Chinese Academy of Science) were cultured in DMEM supplemented with 10% FBS and 1% penicillin/streptomycin at 37°C in a 5% CO_2_ incubator.

### Transfection

Plasmids and 100 mM miRNA mimics or inhibitors (Genepharma, Shanghai, China) were transfected into HEK-293T cells using Lipofectamine 2000 (Invitrogen) according to the manufacturer's protocol. Cells were collected 48 h later for luciferase assays.

### Plasmids construction

The pri-miR-29a, pri-miR-29a/b1 and pri-miR-29b2/c were amplified from mouse genomic DNA by using the following primers and were then cloned into pLKO.1-puro lentiviral vector.

miR-29a F, GGCACCGGTATGCTCGGATGAA GACCTAC;

miR-29a R, GGCGAATTCGGGGCACGTGTTAA TGAAAG;

miR-29a/b1 F, GGCACCGGTACGGACTTCACC TTCCCTCT;

miR-29a/b1 R, GGCGAATTCCAAATCTGCAACC CATACAC;

miR-29b2/c F, GGCACCGGTTGCTCAAAGTGTT GGCTGTA;

miR-29b2/c R, GGCGAATTCGAAGTGATAGG CTGATGCTG.

siRNA oligoes targeting Tet1 were annealed and inserted into the restriction sites of the pLKO.1 lentiviral vector. The siRNA target sequences are as follows: Tet1 KD (5′-GCAGATGGCCGTGACACAAAT-3′). A 1698 bp Tet1 3′-UTR containing 8 predicted miR-29 binding sites were amplified from mouse cDNA with the following primers and was then cloned into the 3′UTR region of the luciferase gene in the psiCheck2 luciferase vector (Promega).

mTet1-UTR F, CTCGAGAGGCTTCTCTC ATGTAATGCC;

mTet1-UTR R, GCGGCCGCCAGAACTCTAA GGCACACAG,

### Mutagenesis

We carried out single point mutations of site-directed mutagenesis by using the Quick-Change Site-Directed Mutagenesis kit (Stratagene). The primers for Tet1 3′-UTR mutagenesis were as follows:

MUT1 F, CATGCTAGAACCATAGAGTCT TTCCCCCGGGTTTGTTTAC;

MUT1 R: GTAAACAAACCCGGGGGAAAGAC TCTATGGTTCTAGCATG;

MUT2 F, GTGTTAACTCTACACAGTTCC TTTAACCACATCAACACAC;

MUT2 R, GTGTGTTGATGTGGTTAAAGGAA CTGTGTAGAGTTAACAC;

MUT3 F, CTGAGAGATCCAAATGCACAATT GCCATTGCTTGGGTTG;

MUT3 R, CAACCCAAGCAATGGCAATTG TGCATTTGGATCTCTCAG;

MUT4 F, CTGTCCTTCAGGGACCCTGATT CTCAGAGATGCCACAAG;

MUT4 R, CTTGTGGCATCTCTGAGAATCA GGGTCCCTGAAGGACAG;

MUT5 F, TTTGTTTTTTTGTTTTTTCCG TTAAAAAGAAAGTCATTC;

MUT5 R, GAATGACTTTCTTTTTAACGGAA AAAACAAAAAAACAAA;

MUT6 F, GAGCTGAGTGATTGTTTTCT CATTGCTCAAGCCTCTTC;

MUT6 R, GAAGAGGCTTGAGCAATGAG AAAACAATCACTCAGCTC;

MUT7 F, GCCCACAACTACCACAATC TAAATGTAAGCCGTTGCAG;

MUT7 R, CTGCAACGGCTTACATTTAGA TTGTGGTAGTTGTGGGC;

MUT8 F, AACTTATTAAACACAATACAAA AGTGTCAGCCTCTGAC;

MUT8 R, GTCAGAGGCTGACACTTTTG TATTGTGTTTAATAAGTT.

### Dual luciferase reporter assay

The psiCheck2 luciferase vector containing wild-type or mutant Tet1 3′-UTR was co-transfected with miR-29a or miR-29b or miR-29c or NC mimics or inhibitors (Genepharma), or pre-miR-29a or pre-miR-29a/b1 or pre-miR-29b2/c or pLKO.1 plasmid into HEK-293T cells, as described earlier. After 48 hours, luciferase activity was determined as an average of the three independent assays using the Dual-Luciferase Reporter assay system (Promega), according to the manufacturer's instructions.

### Lentivirus package

Lentivirus particles were produced by the calcium phosphate transfection according to the method previously described [56]. Briefly, HEK-293FT cells were cultured and co-transfected with generated lentiviral vectors and another two helper vectors psPAX2 and pMD2.G. The medium was replaced 16 hours later. The supernatant was harvested 48 hours and 72 hours after transfection, and filtered through a 0.45 μm filter, then concentrated by precipitation with PEG-8000 (Sigma). The resulting pellet was re-suspended in PBS, aliquoted and frozen at −80°C until use.

### Quantitative reverse transcription-PCR

Total RNA was extracted using Trizol reagent (Invitrogen) according to the manufacturer's protocol. RNA (1 μg) was used for cDNA synthesis using FastQuant RT kit (Tiangen). Real-time PCR was then performed using SYBR premix Ex Taq™ II kit (Takara) in an ABI 7500 real-time PCR cycler. miRNAs were transcribed and qPCR analyzed with PrimeScriptmiRNAqPCR Starter Kit Ver.2.0 (TAKARA). Sequences ofthe primers are described as follows:

TBP (Forward: AGAACAATCCAGACTAG CAGCA; Reverse: GGGAACTTCACATCACAGCTC);

Tet1 (Forard: GAGCCTGTTCCTCGATGTGG; Reverse: CAAACCCACCTGAGGCTGTT);

Tet2 (Forward: TGTTGTTGTCAGGGTGAG AATC; Reverse: TCTTGCTTCTGGCAAACTTACA);

Tet3 (Forward: CCGGATTGAGAAGGTCATCTAC; Reverse: AAGATAACAATCACGGCGTTCT);

Brachyury (Forward: CTGGGAGCTCAGTTC TTTCGA; Reverse: GAGGACGTGGCAGCTGAGA);

Cdx2 (Forward: GTGCGAGTGGATGCGGAAGC; Reverse: CTCCTTGGCTCTGCGGTTCT);

Dlx2 (Forward: CGGACAAGGAAGACCTTGAG; Reverse: GGAGTAGATGGTGCGTGGTT);

Gata4 (Forward: TCCTACTCCAGCCCCTACC; Reverse: GTAGTGTCCCGTCCCATCTC);

Gata6 (Forward: GAGCTGGTGCTACCAAGAGG; Reverse: TGCAAAAGCCCATCTCTTCT);

Hand1 (Forward: CACCAAGCTCTCCAAGATCA; Reverse: GCGCCCTTTAATCCTCTTCT);

Mixl1 (Forward: AGTTGCTGGAGCTCGTCTTC; Reverse:AGGGCAATGGAGGAAAACTC);

Sox7 (Forward: AGATGCTGGGAAAGTCATGG; Reverse: AGAGGGAGCTGAGGAGGAAG);

Sox17 (Forward: GGTCTGAAGTGCGGTTGG; Reverse: TGTCTTCCCTGTCTTGGTTGA).

### Western blotting

Cells were harvested at different time points according to the experimental procedures and lysed for 30 minutes in freshly prepared ice-cold RIPA lysis buffer (Beyotime, P0013B) with PMSF. Lysates were then centrifuged for 30 minutes at 12,000 g at 4°C. Protein concentrations were measured using the Pierce BCA protein assay kit (Pierce, #23225).Sixty micrograms of protein from each sample was used for SDS-PAGE, and then transferred to PVDF membrane. The membrane was blocked for 1 hour in 5% non-fat milk and incubated with primary antibodies overnight at 4°C. Antibodies were used as follows: Tet1 (Genetex, GTX125888, 1:1000), β-actin antibody (Sigma, A2228, 1:30,000). After being washed with TBST, samples were incubated with peroxidase-conjugated secondary antibodies. The immunoreactions were developed using Super Signal West Dura Extended Duration Substrate (Pierce, #34076), and the signal was quantified by measuring the optical density of the bands.

### Dot blot

Genomic DNA was extracted from cells using Universal Genomic DNA Extraction Kit Ver.3.0 (TAKARA, DV811A). 2-fold serial dilutions of genomic DNA was prepared and denatured in 0.4 M NaOH/10 mM EDTA at 99°C for 5 min, cooled down on ice. DNA was spot denatured on membrane (AmershamHybond-N+) and air-dried. The membrane was washed with 2 × SSC buffer and then UV cross-linked. The membrane was blocked with 5% non-fat milk in TBST for 1 h, and then incubated with anti-5hmC antibody (Active motif, 1:10000) at 4°C overnight. The membrane was washed 3 times with TBST for 10 min, and then incubated with HRP secondary antibody for 2 hours at room temperature. The membrane was washed with TBST three times for 10 min, then DNA was detected with ECL by Western blotting analysis system.

### Statistical analysis

Results were presented as the mean ± SEM. Statistical significance was determined using student's *t*-test or one-way ANOVA. The results were considered significant when *p*-value was less than 0.05.
